# Dimethyloxallyl glycine-loaded mesoporous bioactive glass/poly(D,L-lactide) composite scaffolds with ultrasound stimulation for promoting bone repair

**DOI:** 10.3389/fbioe.2024.1339135

**Published:** 2024-02-23

**Authors:** Lei Han, Chaoqian Zhao, Yufang Zhu, Huang Li

**Affiliations:** ^1^ Department of Orthodontics, Nanjing Stomatological Hospital, Affiliated Hospital of Medical School, Institute of Stomatology, Nanjing University, Nanjing, China; ^2^ State Key Laboratory of High Performance Ceramics and Superfine Microstructure, Shanghai Institute of Ceramics, Chinese Academy of Science, Shanghai, China; ^3^ Center of Materials Science and Optoelectronics Engineering, University of Chinese Academy of Sciences, Beijing, China

**Keywords:** scaffolds, mesoporous bioactive glass, dimethyloxalyl glycine, ultrasound stimulation, bone repair

## Abstract

**Introduction:** Bone tissue engineering is considered the ideal approach for bone repair. Mesoporous bioactive glass (MBG) possesses the characteristics of high drug-loading capacity and bioactivity. Low-intensity pulsed ultrasound contributes to promoting fracture healing and bone defect repair, and dimethyloxalyl glycine (DMOG) is a small molecular inhibitor that can suppress prolyl hydroxylase, reducing the degradation of hypoxia-inducible factor.

**Methods:** In this study, we proposed to prepare DMOG-loaded MBG/poly(D,L-lactide) composite scaffolds (DMOG-MBG/PDLLA) for promoting bone repair. The effects of ultrasound stimulation and DMOG release on the cell responses of rat bone marrow mesenchymal stem cells (BMSCs) and human umbilical vein endothelial cells (HUVECs) and bone repair *in vivo* were investigated.

**Results and Discussion:** The results showed that both ultrasound stimulation and DMOG release could promote the proliferation, adhesion and differentiation of BMSCs and HUVECs, respectively. After the implantation of scaffolds in rat cranial bone defect model for 8 weeks, the results indicated that the combined ultrasound stimulation and DMOG release contributed to the highest ability for promoting bone repair. Hence, the DMOG-MBG/PDLLA scaffolds with ultrasound stimulation are promising for application in bone repair.

## 1 Introduction

Bone defects can result from various factors, including infection, tumors, trauma, and congenital diseases (Parsons and Strauss, 2004). Repairing these defects is crucial in preventing pathologic fractures, and several strategies, such as autologous or allogeneic bone grafting, artificial bone implantation, and bone tissue engineering, have been developed for applications (Porter et al., 2009; Peric Kacarevic et al., 2020; Shu et at., 2023; Shi et al., 2023a). Among them, bone tissue engineering focus on reconstructing damaged tissues by utilizing three-dimensional spatial complexes composed of biomaterials and cells, which is considered an ideal approach for bone tissue repair (Bose et al., 2013; Yang et al., 2014). To fabricate bone tissue engineering scaffolds, mesoporous bioactive glass (MBG) has gained much attention due to its abilities of high drug loading and inducing bone-like apatite deposition (Zhu et al., 2015b; Feng et al., 2023; Yang et al., 2023). Furthermore, combining MBG with biodegradable polymers to form composite scaffolds can enhance mechanical strength and regulate biological properties of the scaffolds (Atkinson et al., 2022; Shi et al., 2022; Atkinson et al., 2023; Ma et al., 2023; Sanchez-Salcedo et al., 2023). For example, Wu et al. demonstrated the enhanced osteogenic ability and mechanical strength by loading dexamethasone into the 3D printed MBG scaffolds with poly(vinyl alcohol) as a binder (Wu et al., 2011).

In recent years, there has been a growing interest in smart stimuli-responsive (SSR) biomaterials. Unlike conventional approaches, SSR biomaterials can respond to applied stimuli or changes by altering the physical or chemical structures of materials or catalyzing biochemical reactions, thereby inducing biological effects on cells and tissues. Stimuli-triggered effects of SSR materials can be activated through various ways, including photo stimulation (such as visible light, ultraviolet (UV) light, and near-infrared (NIR) light), magnetic field (MF), electrical stimulation (ES), ultrasound (US), et al (Wu et al., 2021; Bril et al., 2022; Wei et al., 2022; Zhai et al., 2022; Zhang et al., 2024). Among them, low intensity pulsed ultrasound (LIPUS) has been found to promote fracture healing and bone defect repair (Ying et al., 2012; Harrison and Alt, 2021). Studies reported that LIPUS can affect cell metabolism, gene expression and growth factor secretion, accelerating bone defect repair without causing tissue damage (Han et al., 2022; Wang et al., 2022). It also increased local blood flow, improving tissue perfusion and nutrient transfer to the fracture site, thus facilitating healing (Zhu et al., 2015a). Reher et al. applied ultrasound with an intensity of 15–30 mW/cm^2^ to stimulate human osteoblasts, fibroblasts, and chondrocytes, resulting in a significant increase in the expression of cytokines such as IL-8, bFGF, and VEGF. This, in turn, promoted angiogenesis and tissue healing (Reher et al., 1999).

Dimethyloxalylglycine (DMOG) is a small molecular inhibitor that inhibits prolyl-hydroxylase, reducing the degradation of the hypoxia-inducible factor (HIF-1α) and mimicking hypoxic conditions under normal oxygen levels (Rafique et al., 2021). Previous studies have proved that bone marrow-derived mesenchymal stem cells (BMSCs) pretreated with DMOG could activate the expression of HIF-1α, improving angiogenesis and the bone repair ability in bone defects (Hu et al., 2020a; Chen et al., 2021). However, few studies had discussed about the synergistic effects of LIPUS and DMOG on the BMSCs and human umbilical vein endothelial cells (HUVECs) in the bone repairing process.

Herein, we proposed to fabricate multifunctional bone repair scaffolds by 3D printing. The scaffolds were composed of DMOG-loaded MBG and poly(D,L-lactide) (PDLLA), which could promote bone repair through ultrasound stimulation and DMOG release. The effects of ultrasound stimulation and DMOG release on cell adhesion, proliferation, and differentiation of BMSCs and human umbilical vein endothelial cells (HUVECs), and bone repair capability of the scaffolds in rat skull defect model were systematically investigated.

## 2 Materials and methods

### 2.1 Materials

Tetraethyl orthosilicate (TEOS, 98.0%), ethanol (C_2_H_5_OH), triethyl phosphate (TEP) and hydrochloric acid (HCl) were purchased from Group Chemical Reagent Co., Ltd. (Shanghai, China). Calcium nitrate tetrahydrate (Ca(NO_3_)_2_·4H_2_O, 98%) and manganese nitrate tetrahydrate (Mn(NO_3_)_2_·4H_2_, 98%) were obtained from Aladdin Biochemical Technology Co., Ltd. (Shanghai, China). Pluronic P123 (Mn∼5,800), poly(D,L-lactide) (PDLLA, 98.0%) and dimethyloxalyl glycine (DMOG, ≥98%) were purchased from Sigma-Aldrich (United States).

### 2.2 Preparation of DMOG-loaded MBG and DMOG-MBG/PDLLA scaffolds

#### 2.2.1 Preparation of MBG

To prepare the precursor solution, 8.0 g of P123 was dissolved in 200 mL of deionized water and stirred until clear. 22.3 g of TEOS was added to 15 mL of aqueous nitric acid solution with pH = 1, and stirred at room temperature for 1 h. The two solutions were mixed quickly and stirred magnetically for 1 h. Then, 2.44 g of TEP was added to the mixed solution and stirred magnetically for 30 min. Subsequently, 3.79 g of calcium nitrate tetrahydrate and 1.0 g of manganese nitrate tetrahydrate were added sequentially and stirred magnetically for 20 min to obtain the precursor solution.

For the preparation of MBG, a spray drying method was employed (Hu et al., 2020b). The process parameters of the spray dryer (QFN-LE-5, Qiaofeng, Shanghai) were set as follows: the inlet temperature at 220°C, the peristaltic pump speed at 20% (6.67 mL/min), the spray duration of 3 s, and the fan speed at 100%. Once the temperature of the inlet port reached 220°C, the feeding process was initiated. After collecting the powders, they were then calcinated by heating at a rate of 2°C/min until reaching 700°C, held at that temperature for 5 h, and naturally cooled to room temperature to obtain MBG.

#### 2.2.2 Loading of DMOG in MBG

DMOG was loaded in MBG at a ratio of 1:0.035 (MBG: DMOG). After mixing DMOG and MBG, deionized water was added to form a slurry. Then, the slurry was placed under a negative pressure of −0.1 MPa in a vacuum drying oven to remove deionized water for loading DMOG. Finally, the resulting DMOG-loaded MBG (DMOG-MBG) microspheres were freeze-dried for further use.

#### 2.2.3 Preparation of DMOG-MBG/PDLLA scaffolds

Before 3D printing of scaffolds, a slurry was prepared by mixing DMOG-MBG and PDLLA in methylene chloride (CH_2_Cl_2_), and a final mass ratio of MBG: PDLLA: CH_2_Cl_2_: DMOG was about 7:3:10:0.25. After the slurry was transferred to a cylinder, the cylinder was fixed to a 3D printer (BioScaffolder 2.1, GeSiM, Germany). The 3D printing parameters were adjusted as follows: air pressure of 0.4 MPa, a printing layer thickness of 0.6 mm, a printing speed of 10 mm/s, and a needle extrusion line width of 0.6 mm/s. The scaffolds were deposited on a PTFE plate and removed after initial hardening. Finally, the DMOG-MBG/PDLLA scaffolds were obtained after lyophilized with a freeze dryer. The preparation of MBG/PDLLA scaffolds was similar to DMOG-MBG/PDLLA scaffolds, but MBG was without DMOG loading.

#### 2.2.4 Characterization

Scanning electron microscopy (SEM, S-4800, Hitachi, Japan) was utilized for observation of the MBG microspheres and the scaffolds. The MBG microspheres before and after DMOG loading were analyzed using X-ray diffraction (XRD, Bruker D8 Advance, Germany). The specific surface area and mesoporous structure of the MBG microspheres were determined by a specific surface area and pore size analyzer (Quadrasorb SI, United States).

Blue indigo is used to deduce the release of DMOG due to the difficulty in directly testing DMOG. The indigo and DMOG in equal amounts are loaded into mesoporous bioglass, and then fabricated into scaffolds by 3D printing. Before the release experiment, the concentration-absorbance standard curve of indigo was achieved. Then, the scaffold was immersed in PBS solution for 1, 3, 7, 14, 21 days, and the absorbance at different preset times was measured using a spectrophotometer. Finally, the DMOG release was deduced by calculating the indigo release.

### 2.3 Cell culture

For the *in-vitro* cellular experiments, BMSCs and HUVECs were used. BMSCs were cultured in MEM-ALPHA medium (C3060, VivaCell) supplemented with 10% fetal bovine serum (FBS, Gibco) and 1% penicillin-streptomycin mixture (ABT920, G-CLONE). HUVECs were cultured using ECM medium (ScienCell, United States). The cells were incubated with a temperature of 37°C, 5% CO_2_ and 95% humidity. The culture medium was replaced every 2–3 days.

Different groups were established based on the experimental conditions, including the blank group (without the scaffolds, DMOG and ultrasound stimulation), the MBG/PDLLA group (the MBG/PDLLA scaffolds without ultrasound stimulation), the DMOG-MBG/PDLLA group (the DMOG-MBG/PDLLA scaffolds without ultrasound stimulation), the MBG/PDLLA + US group (the MBG/PDLLA scaffolds with ultrasound stimulation), and the DMOG-MBG/PDLLA + US group (the DMOG-MBG/PDLLA scaffolds with ultrasound stimulation).

#### 2.3.1 Cell proliferation

Cell proliferation was determined using the CCK-8 assay (CCK-8, Dojindo, Japan). The scaffolds used were sterilized by UV irradiation for 2 h, and then were transferred to 48-well plates and washed with sterile PBS buffer three times. Typically, 1 × 10^4^ BMSCs or HUVECs cells were seeded in each well and cultured for 1, 3, and 5 days. Ultrasound stimulation was performed by applying stimulation with a power of 0.5 W/cm^2^ for 5 min to the corresponding groups on a daily basis. Following the incubation period, the CCK-8 working solution, diluted 10-fold with fresh MEM-ALPHA medium, replaced the medium in each well and incubated for 2 h. Then, the CCK-8 assay solution was transferred to a new 96-well plate, and the optical density (OD) value was measured at 450 nm using a microplate reader (Epoch, BioTek, Winooski, VT, United States).

#### 2.3.2 Cell adhesion

The scaffolds used for cell adhesion experiments were sterilized by UV irradiation for 2 h, transferred to 48-well plates, and washed with sterile PBS buffer three times. Then, 1ⅹ10^5^ BMSCs or HUVECs cells were seeded on the scaffolds in each well and cultured for 3 days, with daily medium changes. Ultrasound stimulation conditions involved applying sonication with a power of 0.5 W/cm^2^ for 5 min to the corresponding groups on a daily basis. After the predetermined time, the cells were fixed with 4% paraformaldehyde after removed the culture medium. The nuclei and cytoskeleton were stained using 4′,6-diamidino-2-phenylindole dihydrochloride (DAPI) and phalloidin fluorescein isothiocyanate (FITC), respectively. Finally, the adhesion of cells on the scaffolds was observed using a confocal laser scanning microscope (Leica, TCS SP8, Germany).

#### 2.3.3 Cell migration

HUVECs cells were seeded and cultured in a 6-well culture plate at a density of 5ⅹ10^5^ cells per well. Each well was supplemented with 1.5 mL of ECM medium, and the sterilized scaffolds were placed onto Transwells at a ratio of 3 scaffolds per well. After the cells and scaffolds were incubated for 12 h, a line was scribed evenly and straightly in the 6-well plate using a scribing tool, aiming for consistency. The cells were washed with PBS and taken photos to record the cell delineation. Ultrasound stimulation with a power of 0.5 W/cm^2^ was applied to the corresponding groups for 5 min. The cells were further cultured for 12 h, and washed twice with PBS and fixed using 2.5% glutaraldehyde. Finally, the cells were stained using a 0.1% crystal violet solution for 5–10 min, and followed by capturing photos using a light microscope.

#### 2.3.5 Cell differentiation

BMSCs and HUVECs cells were seeded in a 6-well plate at a density of 5ⅹ10^5^ cells per well attaching for 2 h. The sterilized scaffolds were placed on Transwells at a ratio of 3 scaffolds per well, and the cells were incubated (HUVECs for 3 days and BMSCs for 7 days). Ultrasound stimulation with a power of 0.5 W/cm^2^ was applied to the corresponding groups for 5 min daily. After the predetermined time, the plates were washed three times with PBS. Cells were lysed, and RNA was extracted using Trizol reagent. cDNA was synthesized using the reverse transcription kit. The cDNA served as a template for gene-specific RT-qPCR experiments using the SYBR Green PCR Master Mix kit. Gene expression was calculated using the Ct (2^−ΔΔCt^) method.

### 2.4 *In vivo* evaluation of the scaffolds

The animal experiment was approved by the Institution of the Animal Research Welfare and Ethics Committee of Nanjing University (IACUC-D2102055). Cranial bone defect model was created in rats (SPF grade SD rats, healthy, 6–8 weeks old, male, weighing between 180 g and 200 g) to evaluate the regeneration of bone tissue. The rats were divided into five groups, including the blank group, MBG/PDLLA group, DMOG-MBG/PDLLA group, MBG/PDLLA + US group, and DMOG-MBG/PDLLA + US group. Two round and full-layer osteochondral bone defects with a diameter of 5 mm were created on both sides of the mid-cranial suture for each rat, and the scaffolds were implanted into the bone defects. The rats in the MBG/PDLLA + US group and DMOG-MBG/PDLLA + US group received ultrasound stimulation once a week for 5 min, utilizing an ultrasound power of 1 W/cm^2^.

At 8 weeks postoperatively, the rats were euthanized individually. The cranial bone samples, including the periosteum, were taken from the defect site and preserved in paraformaldehyde solution. The surface of the samples was cleaned and fixed in a 4% paraformaldehyde solution for 48 h. The samples were washed and photographed. Furthermore, Micro-CT analysis were performed to evaluate the regeneration of bone tissue.

### 2.5 Statistical analysis

The obtained data were expressed as mean ± standard deviation (SD) based on three or more independent experiments. The statistical significance was analyzed according to a Student’s t-test: *p < 0.05, **p < 0.01, or ***p < 0.001.

## 3 Results and discussion

**FIGURE 1:**
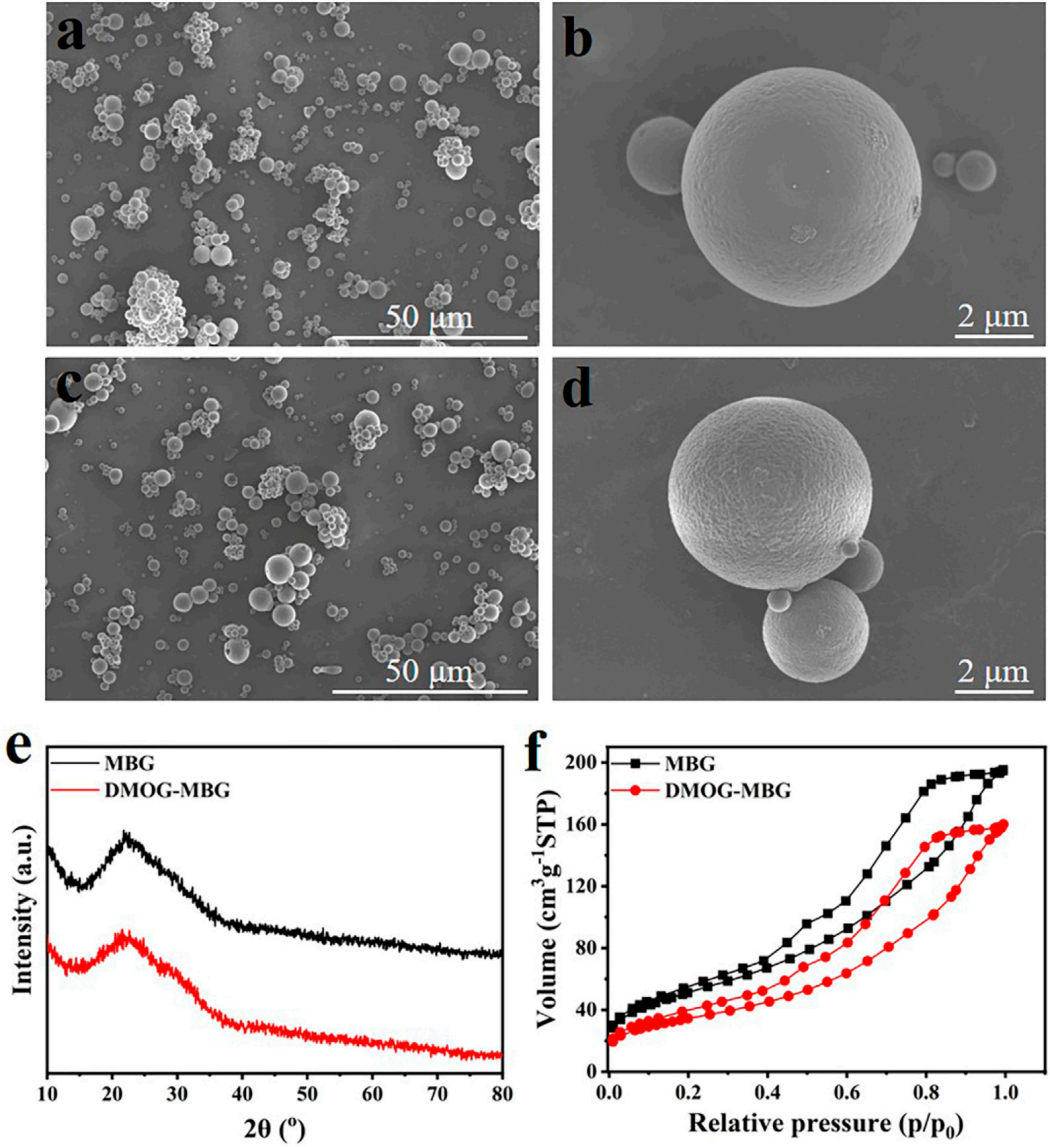
**(A–D)** SEM images, **(E)** XRD patterns and **(F)** nitrogen adsorption-desorption isotherms of MBG and DMOG-MBG microspheres (**A,B** for MBG, and **C,D** for DMOG-MBG).

Figure 1 illustrates SEM images, XRD patterns, and nitrogen adsorption-desorption isotherms of MBG microspheres both before and after the loading of DMOG. It was evident from the images that both the MBG microspheres, before and after DMOG loading, exhibited a high degree of sphericity with a particle size ranging from 1 to 5 μm (Figures 1A–D). This indicated that the loading of DMOG did not have an impact on the microspheres’ morphology. The XRD analysis confirmed that the MBG microspheres were amorphous, and no new diffraction peaks were observed following the loading of DMOG (Figure 1E). This observation suggested that DMOG, when loaded into the mesopore channels of the MBG microspheres, existed in an amorphous state, likely due to the confinement effect of the mesopore channels. Analysis of nitrogen adsorption-desorption revealed a noticeable reduction in nitrogen adsorption for DMOG-loaded MBG microspheres compared to the unmodified MBG microspheres (Figure 1F). Nevertheless, the shape of the isotherm hysteresis loop remained unchanged, indicating that the DMOG-MBG microspheres maintained their original mesoporous structure, albeit with a reduced specific surface area. According to the BET (Brunauer–Emmett–Teller) method, the specific surface areas of MBG and DMOG-MBG microspheres were approximately 187.1 m^2^/g and 125.2 m^2^/g, respectively, confirming the successful loading of DMOG into the mesopores of the MBG microspheres.

**FIGURE 2:**
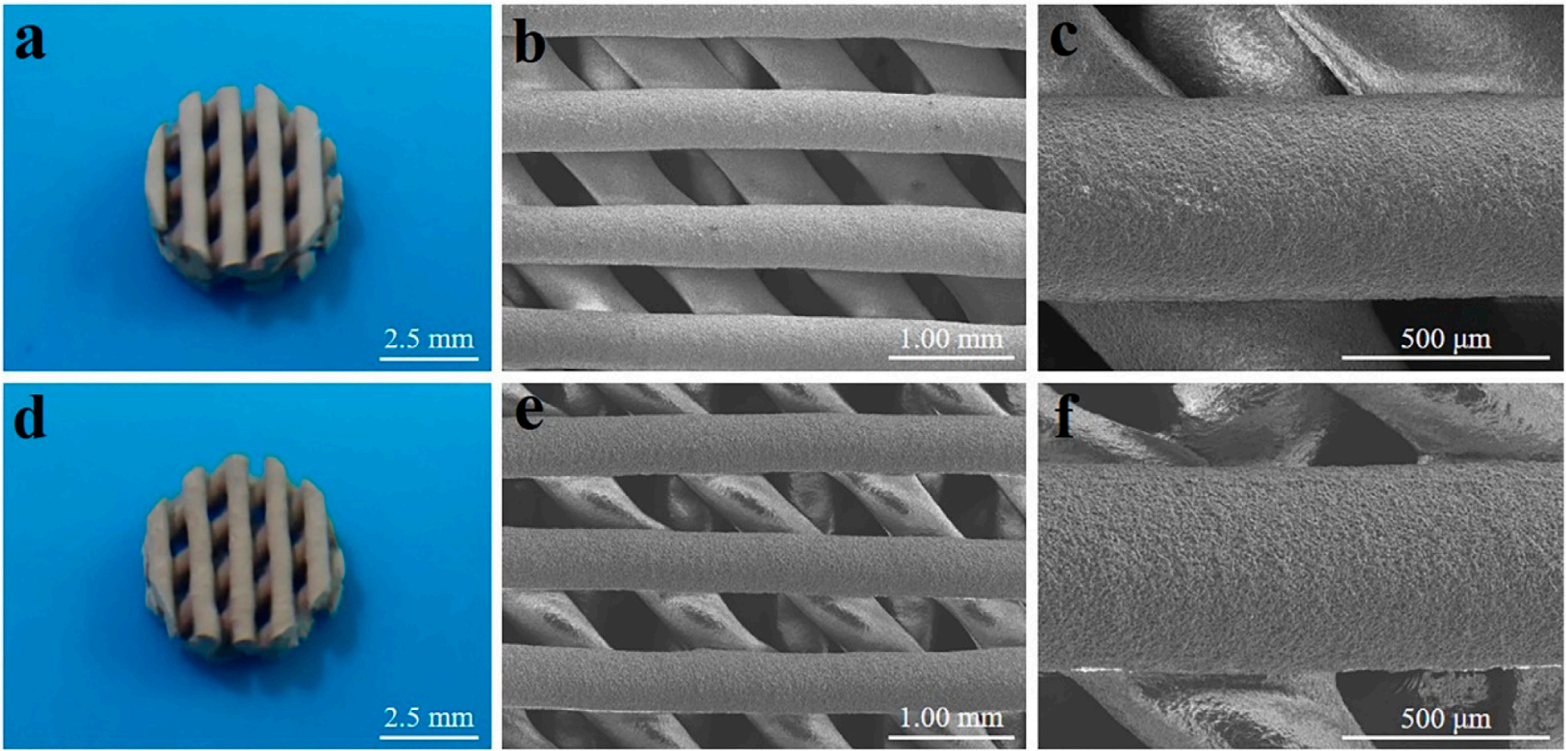
Photographs and SEM images of **(A–C)** MBG/PDLLA and **(D–F)** DMOG-MBG/PDLLA scaffolds.

**FIGURE 3:**
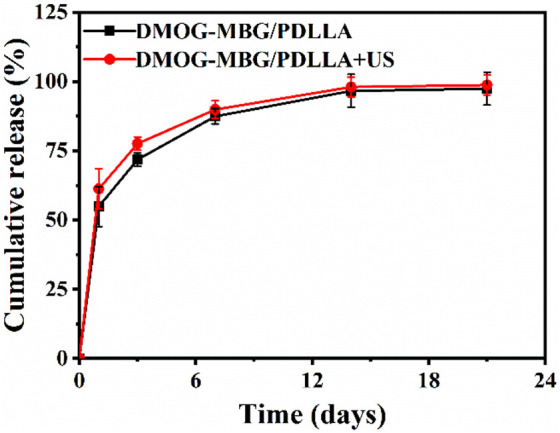
The cumulative DMOG release profiles of DMOG-MBG/PDLLA scaffolds with and without ultrasound stimulation.

Optical photographs and scanning electron microscope (SEM) images of MBG/PDLLA and DMOG-MBG/PDLLA scaffolds are shown in Figure 2. Both scaffolds exhibited a similar and consistent macroporous structure, indicating that the loading of DMOG in MBG did not affect the printing performance of the scaffolds. Figure 3 illustrates the cumulative release of DMOG from the DMOG-MBG/PDLLA scaffolds with and without ultrasound stimulation. It demonstrated a sustained release behavior of DMOG from the DMOG-MBG/PDLLA scaffolds, with ultrasound stimulation enhancing DMOG release to a certain extent. The accelerated release of DMOG through ultrasound stimulation might yield beneficial effects on cell responses.

**FIGURE 4:**
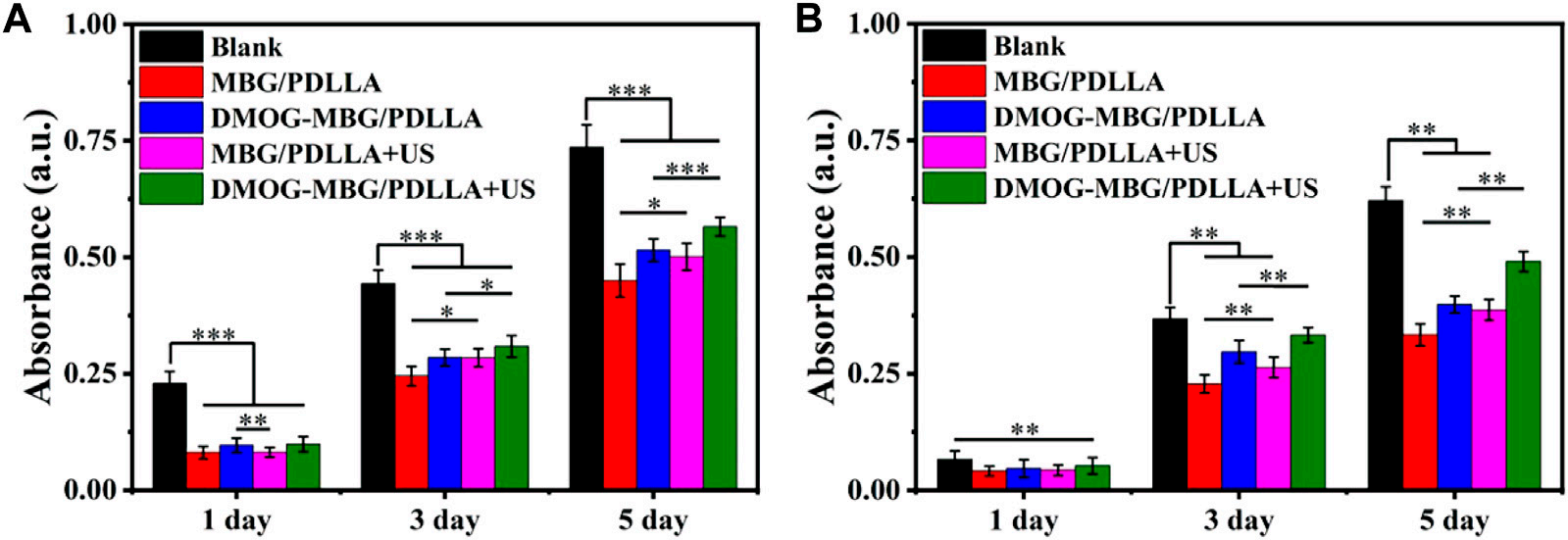
The proliferation of **(A)** HUVECs and **(B)** BMSCs on the MBG/PDLLA and DMOG-MBG/PDLLA scaffolds with and without ultrasound stimulation.

The effects of the scaffolds and ultrasound stimulation on the proliferation of HUVECs are shown in Figure 4A. It can be found that the blank group displayed the highest cell growth, while the MBG/PDLLA and DMOG-MBG/PDLLA scaffold groups showed slightly lower cell growth, with or without ultrasound stimulation. However, as the incubation time extended, cell proliferation became more noticeable, underscoring the cytocompatibility of both the MBG/PDLLA and DMOG-MBG/PDLLA scaffolds. Specifically, there was no significant difference in cell growth between the scaffold groups with or without ultrasound stimulation on day 1. However, by day 3, cell proliferation among the groups followed this order: MBG/PDLLA < DMOG-MBG/PDLLA ≈ MBG/PDLLA + US < DMOG-MBG/PDLLA + US, with a significant difference observed between the DMOG-MBG/PDLLA and MBG/PDLLA groups. Similarly, the trend in cell proliferation at day 5 mirrored that of day 3, with the DMOG-MBG/PDLLA + US group exhibiting statistically significant differences compared to the other groups. These results suggested that the release of DMOG promoted HUVEC proliferation, and ultrasound stimulation also had a positive impact on cell proliferation.

**FIGURE 5:**
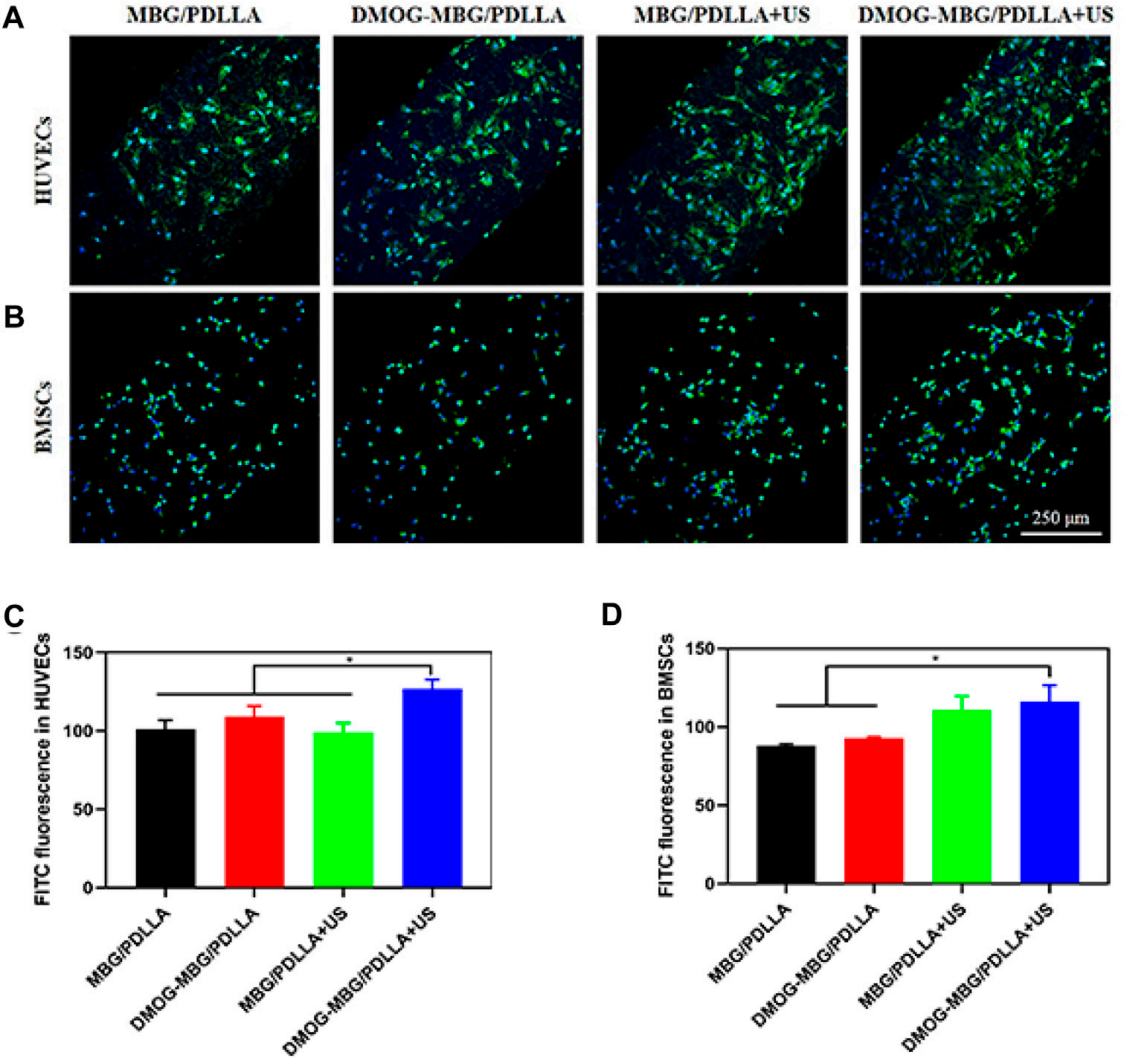
The adhesion of **(A, C)** HUVECs and **(B, D)** BMSCs on the MBG/PDLLA and DMOG-MBG/PDLLA scaffolds with and without ultrasound stimulation.

Furthermore, the adhesion and spreading of HUVECs on the MBG/PDLLA and DMOG-MBG/PDLLA scaffolds after 3 days of culture were observed to be good, with or without ultrasound stimulation (Figures 5A,C), which indicated the biocompatibility of both types of scaffolds. The DMOG-MBG/PDLLA + US group demonstrated the best cell spreading than the other three groups under FITC fluorescence observation.

**FIGURE 6:**
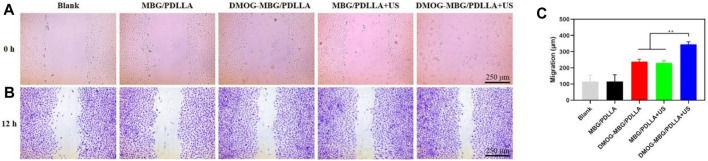
The effects of the scaffolds and ultrasound stimulation on the migration of HUVECs after cultured for 12 h, **(A)** 0 hours **(B)** 12 hours and **(C)** quantitative analysis of migration.

Additionally, as shown in Figure 6, the cell migration results aligned with the cell proliferation findings, with HUVEC migration ranked as follows: Blank < MBG/PDLLA < DMOG-MBG/PDLLA ≈ MBG/PDLLA + US < DMOG-MBG/PDLLA + US. In summary, both ultrasound stimulation and the release of DMOG promoted HUVEC proliferation, adhesion, and migration.

**FIGURE 7:**
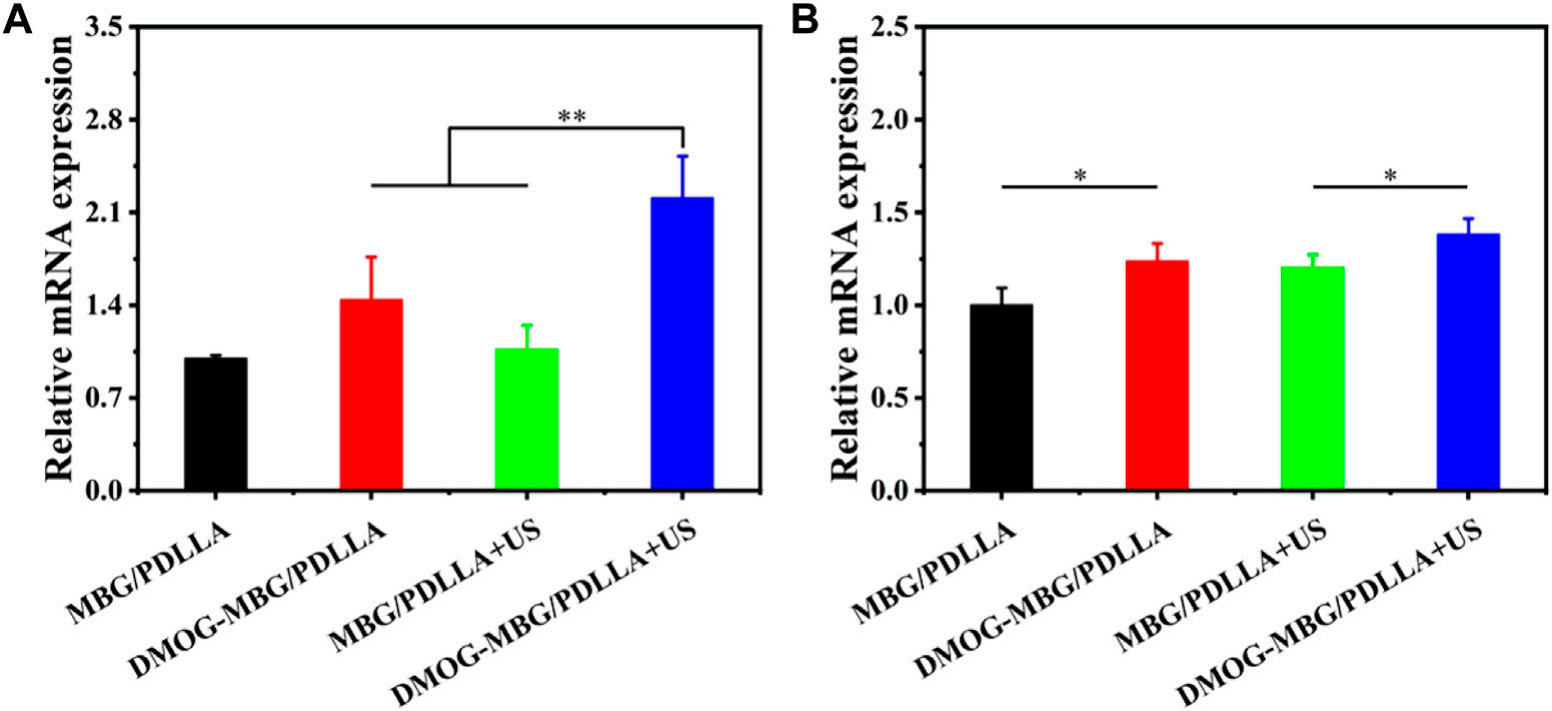
The expression of angiogenesis-related genes, **(A)** HIF-1α and **(B)** VEGF, of HUVECs cultured with the MBG/PDLLA and DMOG-MBG/PDLLA scaffolds for 3 days with and without ultrasound stimulation.

Figure 7 presents the impact of the scaffolds and ultrasound stimulation on the differentiation of HUVECs, focusing on the expression of angiogenesis-related genes, HIF-1α and VEGF. Notably, the DMOG-MBG/PDLLA + US group exhibited the highest expression of HIF-1α, and this expression level was significantly different from the other three groups. The expression of VEGF across the different groups followed this order: MBG/PDLLA < DMOG-MBG/PDLLA ≈ MBG/PDLLA + US < DMOG-MBG/PDLLA + US, with statistically significant differences observed between the MBG/PDLLA group and the other three groups, as well as between the MBG/PDLLA + US and DMOG-MBG/PDLLA + US groups. These results provided compelling evidence that both ultrasound stimulation and the release of DMOG could actively promote the expression of angiogenesis-related genes, thereby facilitating the process of vascularization.

The effects of the scaffolds and ultrasound stimulation on the proliferation of BMSCs are shown in Figure 4B. On day 1, no statistically significant differences were observed in cell proliferation among the various groups, including MBG/PDLLA and DMOG-MBG/PDLLA scaffolds, both with and without ultrasound stimulation. However, by day 3, cell proliferation was notably higher in the DMOG-MBG/PDLLA and MBG/PDLLA + US groups when compared to the MBG/PDLLA group, and the DMOG-MBG/PDLLA + US group exhibited further enhanced cell proliferation. This trend in BMSC proliferation on day 5 remained consistent with the observations made on day 3. Furthermore, as shown in Figures 5B,D, both the MBG/PDLLA and DMOG-MBG/PDLLA scaffold groups, with or without ultrasound stimulation, supported the adhesion of BMSCs. Among these groups, the DMOG-MBG/PDLLA + US group demonstrated the best cell spreading. Therefore, these results underscore that both ultrasound stimulation and the release of DMOG actively promote the proliferation and adhesion of BMSCs.

**FIGURE 8:**
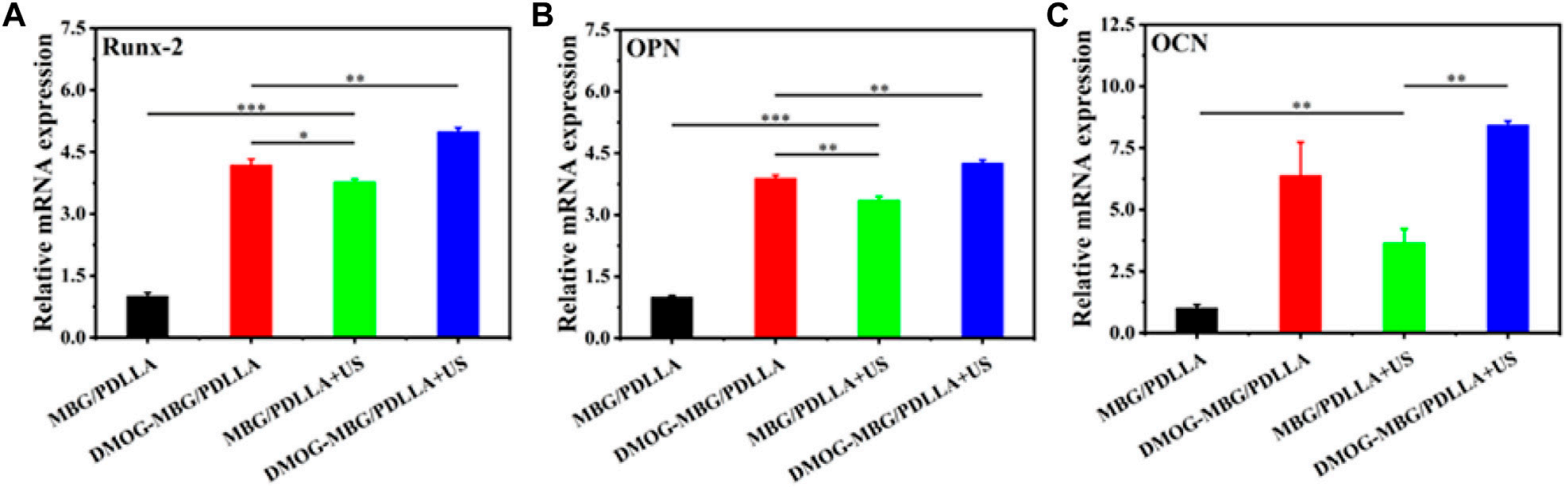
The effects of the scaffolds and ultrasound stimulation on the differentiation of BMSCs after cultured for 7 days. The gene expression **(A)** Runx-2 **(B)** OPN and **(C)** OCN.

Figure 8 shows the effects of the scaffolds and ultrasound stimulation on the differentiation of BMSCs after they were cultured for 7 days. By analyzing the expression of osteogenic-related genes in BMSCs, such as Runx2, OPN, and OCN, it was observed that the expression of osteogenic-related genes significantly increased in the DMOG-MBG/PDLLA, MBG/PDLLA + US group, and DMOG-MBG/PDLLA + US groups compared to the MBG/PDLLA group. Moreover, the DMOG-MBG/PDLLA + US group exhibited the highest expression level. Therefore, it can be concluded that ultrasound stimulation and DMOG release can promote the differentiation of BMSCs individually, and the combined ultrasound stimulation and DMOG release further enhance the differentiation of BMSCs.

**FIGURE 9:**
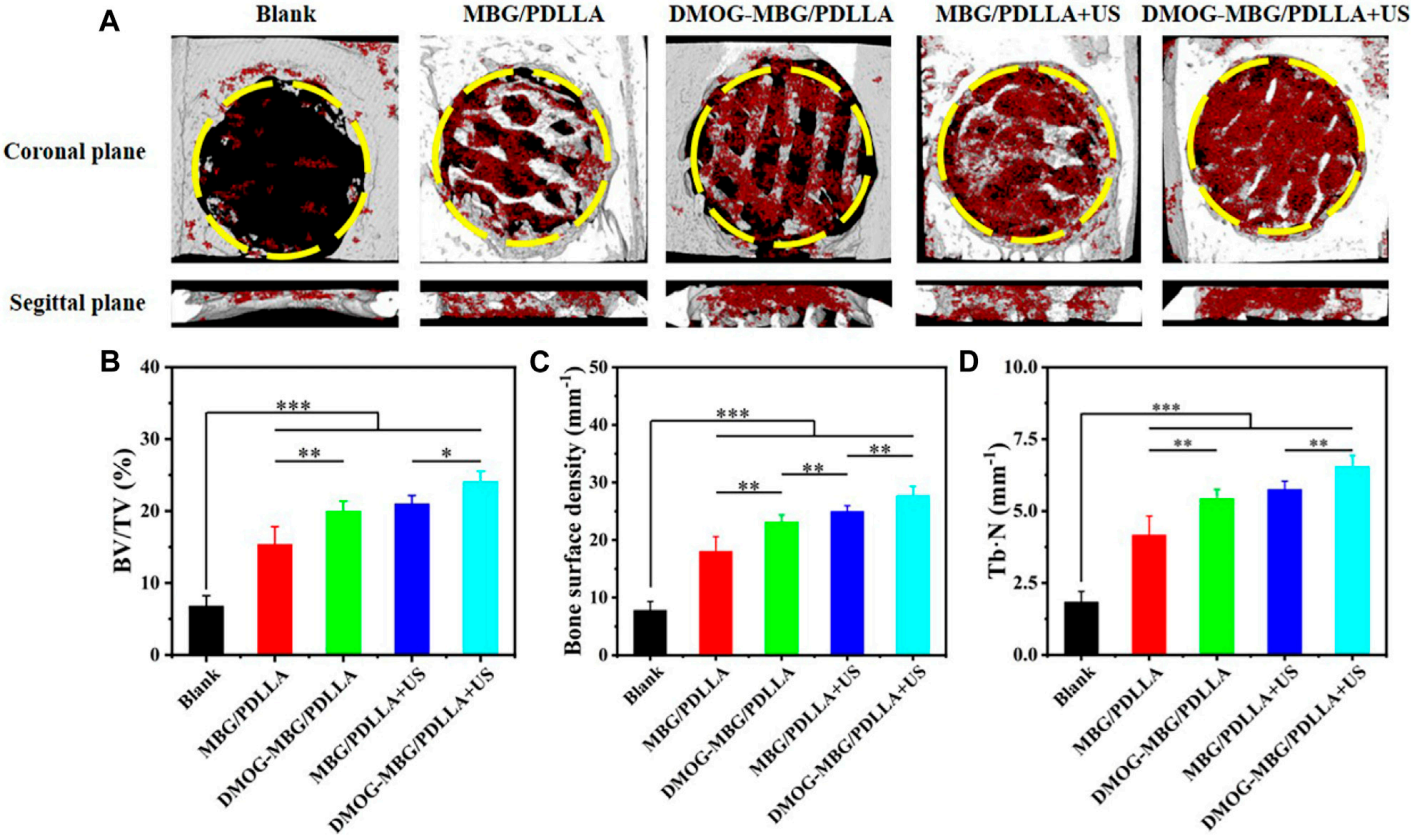
*In vivo* bone repair effect of the scaffolds by implanting them into rat cranial bone defects after 8 weeks **(A)** the coronal planes and sagittal planes of the bone defects reconstructed by Micro-CT. The red color and grey-white color in Micro-CT images represent new bone and scaffold, respectively. The newly formed bone assessment including **(B)** the ratio of new bone volume to defect volume (BV/TV), **(C)** bone surface mineral density and **(D)** the trabecular number of the new bone (Tb.N).

Figure 9 shows the *in vivo* bone repair effect of the scaffolds by implanting them into rat cranial bone defects after 8 weeks. Micro-CT 3D reconstruction images revealed that the scaffold effectively fills the skull defect site and exhibits good integration with the skull. Further analysis of the amount of new bone formation, including new bone volume fraction, bone density, and trabecular number, indicated that the blank group exhibited only a small amount of new bone formation, with no significant changes in the overall defect. The MBG/PDLLA group showed more new bone formation, primarily distributed along the scaffold walls. The amounts of new bone in the DMOG-MBG/PDLLA and MBG/PDLLA + US groups were close to each other but greater than that in the MBG/PDLLA group. Importantly, the DMOG-MBG/PDLLA + US group had the highest new bone formation. Overall, these results suggest that the implantation of the MBG/PDLLA and DMOG-MBG/PDLLA scaffolds promotes the repair of bone defects, and both ultrasound stimulation and DMOG release contribute to enhanced bone repair ability."

## 4 Conclusion

In this study, we prepared DMOG-MBG/PDLLA composite scaffolds for bone repair by 3D printing. The DMOG-MBG/PDLLA scaffolds exhibited sustained DMOG release behavior due to the DMOG loading in mesopores of MBG microspheres, and ultrasound stimulation could accelerate DMOG release in a certain extent. Importantly, both ultrasound stimulation and DMOG release could promote the proliferation, adhesion and differentiation of BMSCs and HUVECs, as well as the migration of HUVECs. *In vivo* evaluation in rat cranial bone defect model indicated that the DMOG-MBG/PDLLA scaffolds promoted the repair of bone defect due to DMOG release, and ultrasound stimulation further improved bone repair ability. Hence, this study demonstrated the positive role of ultrasound stimulation and DMOG release in bone repair, and the DMOG-MBG/PDLLA scaffolds have great potential for bone repair applications.

## Data Availability

The raw data supporting the conclusion of this article will be made available by the authors, without undue reservation.
